# Bergman glial cell morphology under the high voltage Electron microscope

**DOI:** 10.1186/s42649-019-0007-3

**Published:** 2019-07-01

**Authors:** Im Joo Rhyu

**Affiliations:** 1 Department of Anatomy, Korea University College of Medicine, Goryeodae-ro 73 (Anam-dong 5ga), Seongbuk-gu, Seoul, 02841 South Korea; 2 Division of Brain Korea 21 Plus Program for Biomedical Science, Korea University College of Medicine, Seoul, South Korea

**Keywords:** Bergman glial fiber, Golgi epithelial cell, HVEM, Microdomain, Rapid Golgi stain

## Abstract

The detailed morphology of Bergam glial cell was observed in single field of view during observation of Golgi stained mouse cerebellar cortex under the high voltage electron microscopy. The 3-dimensional organization of Bergman glial cell fully demonstrated with 8-degree stereo-paired images. The morphology of Bergman glial fiber and its appendages forming microdomains connected to other glial fiber are clearly presented in this image. This image provides a valuable insight for understanding morphology of Bergman glial cell.

Bergman glial cell (BG) is a peculiar to cerebellar cortex, is also called Golgi epithelial cell. This cell is reported as one of three variants of astrocyte in the cerebellum (Palay and Chan-Palay 1972). The BGs play important role in granule cell migration and cerebellar development. In addition to the role during development, some functions are reported in extracellular ion homeostasis, neuronal protective metabolism and synaptic modulation (De Zeeuw and Hoogland 2015). The overall description on morphology of the BG has been reported based on light microscope with immunohistochemistry or Golgi stain. Although light microscopic observation of Golgi staining BG provides morphological characteristics of the cell, detailed image reported was mainly reported based on camera lucida drawing due to the technical limitation of light microscopy and camera system.

The overall morphology of the BG (Fig. 1) was captured in single field of view under the high voltage electron microscopy (Hitachi H-1250 M, Japan) of 4 micro-thickness sectioned rapid Golgi stained mouse cerebellum originally prepared to study Purkinje cell morphology with rapid Golgi method (Oda et al. 2010)

**Fig. 1:**
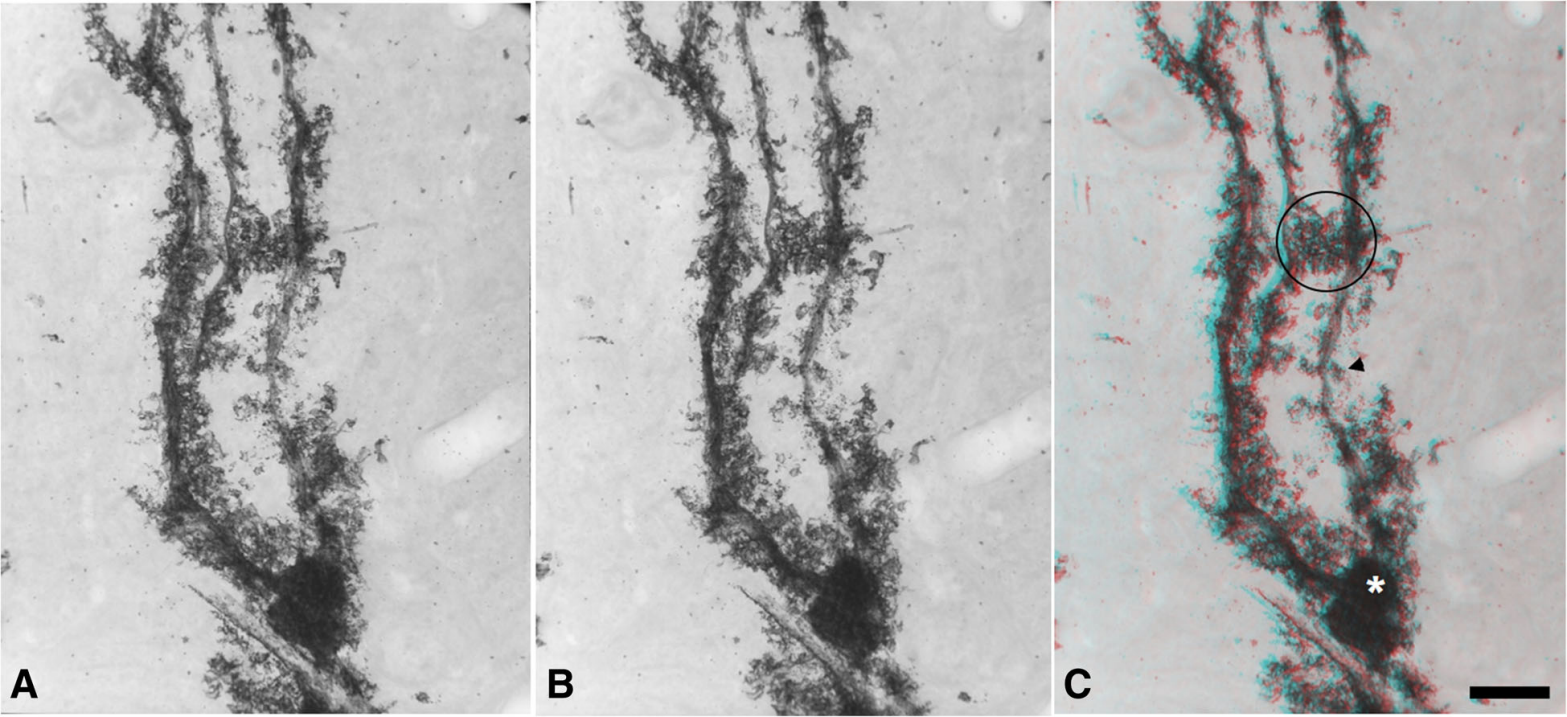
High voltage electron microscopic image of Bergman’s glial cell. The same Bergman’s glial cell was taken at different angles: **a** 0 degree; **b** 8 degree tilted. From this paired image, an anaglyphic image can be constructed (**c**). By examining the blue red image with the use of blue/red stereoscopic glasses, a three dimensional image will be apparent. Cell body (*) and microdomains (circle) were clearly observed on analyphic image (**c**). (Scale bar: 5 µm)

The stereo-pair images (Fig. 1A, B) demonstrate 3-dimensional organization of the Bergman glial cell through molecular layer. If blue and red glasses were put on to observe anaglyph image (Fig. 1C), 3-dimensional perception is easily possible (Scale bar: 5 m).

The cell body (asterisk on Fig. 1C) looks like to be globular shape, and its surface has numerous thorny to lamellar protrusions as Palay described (Palay and Chan-Palay 1972). Bergman glial fibers take off branches from the upper part of the cell body. They run straightly throughout the molecular layer to the pial surface of the cerebellum. The Bergman fibers hold irregular appendages. One type is short thorn processes (Arrow head on Fig. 1C), the other is elaborate processes. These processes are intermingled with neighboring glial fibers to form microdomains (Circle on Fig. 1C), which is supposed to act as compartment of synapses covering in electronically and biochemically (Grosche et al. 1999). Some pores are observed in their lamellar process, which are expected the site traversing neural component such as dendrites and axons of the cerebellar neurons. The BG microdomains are supposed to regulate the activities of the neural components and synapses embedding in them (De Zeeuw and Hoogland 2015). If electron tomogragphic analysis could be applied to Gogi stained BG cells, additional information would be extractable (Hama et al. 2004).

This image provides a valuable insight for understanding morphology and function of Bergman glial cell.

## Data Availability

Not applicable. “Please contact author for data requests.”
